# Weighted persistent homology for osmolyte molecular aggregation and hydrogen-bonding network analysis

**DOI:** 10.1038/s41598-020-66710-6

**Published:** 2020-06-16

**Authors:** D. Vijay Anand, Zhenyu Meng, Kelin Xia, Yuguang Mu

**Affiliations:** 1 Division of Mathematical Sciences, School of Physical and Mathematical Sciences, Nanyang Technological University, Singapore, 637371 Singapore; 2 School of Biological Sciences, Nanyang Technological University, Singapore, 637371 Singapore

**Keywords:** Structural biology, Biochemistry

## Abstract

It has long been observed that trimethylamine N-oxide (TMAO) and urea demonstrate dramatically different properties in a protein folding process. Even with the enormous theoretical and experimental research work on these two osmolytes, various aspects of their underlying mechanisms still remain largely elusive. In this paper, we propose to use the weighted persistent homology to systematically study the osmolytes molecular aggregation and their hydrogen-bonding network from a local topological perspective. We consider two weighted models, i.e., localized persistent homology (LPH) and interactive persistent homology (IPH). Boltzmann persistent entropy (BPE) is proposed to quantitatively characterize the topological features from LPH and IPH, together with persistent Betti number (PBN). More specifically, from the localized persistent homology models, we have found that TMAO and urea have very different local topology. TMAO is found to exhibit a local network structure. With the concentration increase, the circle elements in these networks show a clear increase in their total numbers and a decrease in their relative sizes. In contrast, urea shows two types of local topological patterns, i.e., local clusters around 6 Å and a few global circle elements at around 12 Å. From the interactive persistent homology models, it has been found that our persistent radial distribution function (PRDF) from the global-scale IPH has same physical properties as the traditional radial distribution function. Moreover, PRDFs from the local-scale IPH can also be generated and used to characterize the local interaction information. Other than the clear difference of the first peak value of PRDFs at filtration size 4 Å, TMAO and urea also shows very different behaviors at the second peak region from filtration size 5 Å to 10 Å. These differences are also reflected in the PBNs and BPEs of the local-scale IPH. These localized topological information has never been revealed before. Since graphs can be transferred into simplicial complexes by the clique complex, our weighted persistent homology models can be used in the analysis of various networks and graphs from any molecular structures and aggregation systems.

## Introduction

Tri-methylamine N-oxide (TMAO) and urea are organic osmolytes widely existing in the animal metabolisms. Deep-sea organisms use the protein stabilizing effects of TMAO to counteract the high pressure perturbation, while mammalian kidneys use the strong denaturant function of urea^1^. As a protecting osmolyte, TMAO can counteract the urea protein-denaturing effects. Currently, it is well accepted that urea acts by directly binding to the protein backbones and side chains^2^. It has very little disturbance on the surrounding water structures. The TMAO’s stabilization is not well understood. It has been suggested that TMAO molecules form complexes with two to three water molecules, and protein stabilization is the result of depletion effects associated with unfavorable interaction of TMAO with protein backbone^3^. Others suggest that TMAO interacts with polypeptides and protein stabilization is a result of surfactant-like effects of TMAO^4^. The interaction between TMAO and urea is also not well understood^5^. Even though it is suggested that the interaction is through the TMAO’s modification of urea-water structures, recent experiments show that the addition of TMAO induces blue shifts in urea’s H-N-H symmetric bending modes, indicating the direct interactions between the two cosolvents^6,7^. Although great progress has been made in both experimental and theoretical research for urea and TMAO^8–20^, a detailed mechanism of their molecular aggregations and the corresponding hydrogen-bonding networks still remain elusive.

Theoretically, graph or network based models^21–23^, especially the spectral graph models and combinatorial graph models, play a key role in the characterization of biomolecular structures, interaction networks, and hydrogen-bonding networks^24–30^. The most commonly-used graph-based measurements^31^ include, node degree, shortest path, clique, cluster coefficient, closeness, centrality, betweenness, Cheeger constant, modularity, graph Laplacian, graph spectral, Erdös number and percolation information. Differential geometry tools^32–38^, such as Voronoi diagram, alpha shape, geometric flows, have also been considered to quantitatively characterize the biomolecular structure, surface, volume, cavity, void, tunnel and interface. These models contribute greatly to the better understanding of biological systems.

Recently, a new topological model known as persistent homology has demonstrated a great promise in biomolecular structure, flexibility, dynamics and function analysis^39–41^. Persistent homology based machine learning and deep learning models^42^ have achieved great successes in protein-ligand binding affinity prediction^43–45^, protein stability change upon mutation^46,47^ and toxicity prediction^48^. These topology based machine learning models have constantly achieved some of the best results in D3R Grand challenge^49^. Motivated by the great success of topological modeling in biomolecules, we have applied persistent homology in the analysis of ion aggregations and hydrogen-bonding networks^50^. The two types of ion aggregation models, i.e., local clusters and extended ion networks, can be well characterized by our model. Further, we have identified, for the first time, different types of topology for the two hydrogen-bonding network systems^50^. Moreover, we have studied the osmolyte molecular aggregation and their hydrogen-bonding networks^31^. Two osmolytes, i.e., TMAO and urea, are found to share very similar topological patterns with the two types of ion systems, i.e., KSCN and NaCl. Particularly, the topological fingerprints for the hydrogen-bonding network from ion systems and osmolyte systems share a great similarity. This indicates that our topological representation can characterize certain intrinsic difference between “structure making” and “structure breaking” systems^31^. Features from persistent homology can be used as topological descriptors, which have also been used in a range of atomistic water models^51^, coarse-grained Stillinger-Weber (SW) potential model^51^, and aqueous solubility modeling^52^.

More recently, weighted persistent homology (WPH) models have been proposed to incorporate physical, chemical and biological properties into topological modeling^53^. Essentially, the weight value, which reflects physical, chemical and biological properties, can be assigned to vertices (atom centers), edges (bonds), or higher order simplexes (cluster of atoms), depending on the biomolecular structure, function, and dynamic properties^53^. In this way, weighted persistent homology can be classified into three major categories, i.e., vertex-weighted^54–58^, edge-weighted^41,44,46,59,60^, and simplex-weighted models^61–63^. Among them, the localized (weighted) persistent homology (LPH) and interactive persistent homology (IPH) are found to be of great importance in the classification and clustering of DNA structures and trajectories^53^, and protein ligand interactions^46^.

In this paper, for the first time, we apply the localized persistent homology and interactive persistent homology in the study of osmolyte molecular aggregation and their hydrogen-bonding networks. To quantitatively characterize the topological features from LPH and IPH, we propose Boltzmann persistent entropy (BPE). We have revealed that TMAO and urea have very different local topologies. Local network structures are observed in TMAO system. With the concentration increase, the circles within these networks show a huge increase in their total numbers and a sharp decrease in their relative sizes. In contrast, urea shows two distinguishable local topological features, i.e., local clusters around 6 Å and global-scale circle structures at around 12 Å. Further, we have demonstrated that our global-scale IPH based persistent radial distribution function (PRDF) is similar to the traditional radial distribution function (RDF) and can be used to characterize the double layer information. Moreover, a local-scale PRDFs can be generated from our local-scale IPH model. Essentially, in global-scale IPH, each osmolyte molecule interacts with all the water molecules in the system. In local-scale IPH, water molecules are classified into different cells based on the Voronoi diagram of osmolyte molecules. Interactions only happen between a central osmolyte molecule and the surrounding water molecules within its Voronoi cell and between two osmolyte molecules from closest adjacent Voronoi cells. This classification is naturally embedded in the filtration process of IPH analysis. Further, IPH based PBNs and BPEs can be used in studying the interaction patterns between osmolyte molecules and water molecules. Other than osmolyte systems, our weighted persistent homology models can be applied in the analysis of various kinds of networks and graphs from material, chemical, and biological systems.

The paper is organized as follows. A brief introduction of persistent homology and two weighted persistent homology models are given in Section 1.1 and Section 1.2. The methodology and implementation details of LPH and IPH models are discussed in Section 1.3. The main results are presented in Section 2. The LPH based molecular aggregation and hydrogen-bonding networks is discussed in Section 2.1. The IPH based topological features for osmolyte-water interaction networks are discussed in Section 2.2. The paper ends with a conclusion.

## Methods and models

In this section, we will give a brief introduction of persistent homology and weighted persistent homology. Three types of persistent functions, including persistent Betti number, persistent entropy and persistent radial distribution function, will be discussed in detail. A general description of the two WPH models, i.e., localized persistent homology and interactive persistent homology, will also be presented.

### Persistent homology

The persistent homology, a tool from algebraic topology and computational topology, is proposed to characterize data “shape”^64^. It has been widely used in data analysis^64–81^ with various developed softwares^59,82–87^ and visualization models^88–91^.

Persistent homology can be understood from three different aspects. Firstly, it is the relation between a graph and a simplicial complex. Mathematically, a graph, which is composed of only nodes (0-simplexes) and edges (1-simplexes), is a kind of simplicial complex. A simplicial complex *K* can viewed as a set of simplexes that satisfy two conditions. Firstly, any face of a simplex from *K* is also in *K*. Secondly, the intersection of any two simplexes in *K* is either empty or a shared face^92^. Other than 0- and 1-simplexes, it also includes 2-simplexes (solid triangles), 3-simplexes (tetrahedrons), and other higher-dimensional components. Secondly, it is about geometric measurements and topological invariants. In persistent homology, the data is characterized by Betti numbers, including *β*_0_, *β*_1_, *β*_2_ and higher order topological invariants^93,94^. These measurements are significantly different from previous geometric measurements, like distances, angles, areas, etc. Thirdly, it is the difference between single scale model and multi-scale representation. Essentially, a series of related simplical complexes are considered in persistent homology and they provide a multiscale representation that balances geometry and topology. A more detailed description of its mathematical background can be found in refs. ^93–95^, and its application in molecular biology and ion aggregation systems can be found in refs. ^31,41,44,46,50,52^.

**Figure 1:**
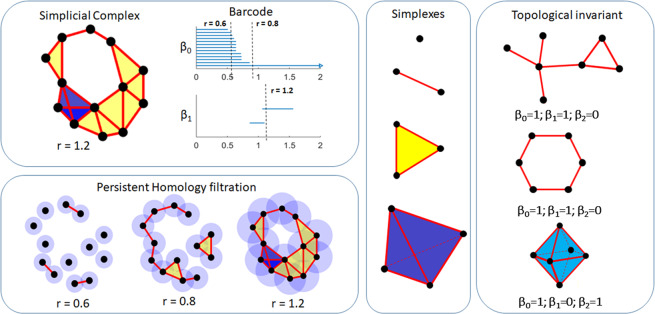
The illustration of the basic components in persistent homology. Essentially, persistent homology is based on simplicial complex, which is composed of simplexes. In persistent homology, only topological invariants, known as Betti number, are considered. A series of simplicial complexes are generated through a filtration process. The results from persistent homology are represented as persistent barcodes.

Geometrically, *β*_0_ indicates the number of connected components, *β*_1_ corresponds to the number of circles, rings or loops, and *β*_2_ represents the number of voids or cavities. The key concept in persistent homology is the filtration^93,94^. For instance, given a point cloud data, we can associate each point with an identical-sized sphere and assign its radius as the filtration parameter. As the filtration value is increased, these spheres will systematically enlarge and subsequently merge with each other to form simplexes. Roughly speaking, an edge between two points is formed when the two corresponding spheres overlap^93,94^. A triangle is formed when each of two spheres (of the three corresponding spheres from triangle vertices) overlap. A tetrahedron is formed when each three spheres (of the four corresponding spheres from tetrahedron vertices) overlap^92^. At each filtration value, all the simplexes, i.e., vertices, edges, triangles, tetrahedrons, form a simplicial complex. From it, topological invariants, i.e., Betti numbers, can be calculated. The persistent homology hierarchically increases the complexity in data representation by systematically incorporating higher order simplices as the filtration proceeds. This enables a multiscale representation of topological invariants from simplicial complexes^93–95^. In this way, a systematic variation of the filtration parameter leads to a series of simplicial complexes at different scales^93–95^. Some topological invariants persist longer in these simplicial complexes, while others disappear quickly as the filtration value is increased. The length of the *β*_1_ bar defines the “lifespan” of the topological invariants (circles, loops, etc) and provides a natural geometric measurement^93–95^. More specifically, the lifespan, known as the persistence, measures how “large” are the circles, loops and voids in the system. We denote a filtration value at which a topological invariant formed or killed as birth time and death time respectively. In this way, each topological invariant has a “lifespan” defined by its birth and death time. Essentially, the lifespan provides a geometric measurement of the topological invariant. If we use a one-dimensional bar, which starts at a birth time and ends at a death time, to represent each homology generator, a barcode representation is generated. Figure 1 illustrates the basic topological components, including simplexes, Betti number, filtration process, and persistent barcodes.

**Figure 2:**
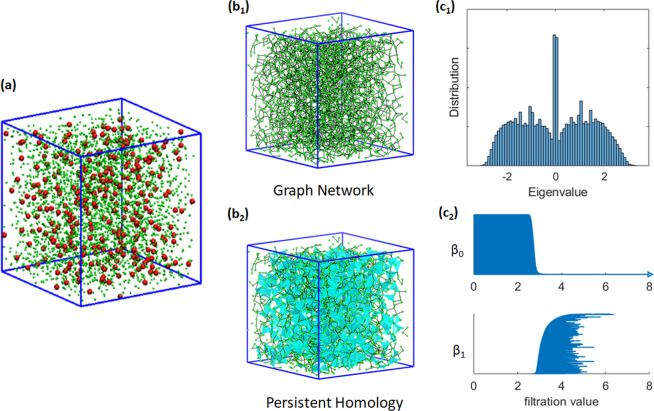
Illustration of the comparison between graph models and persistent homology for osmolyte molecular aggregation. (**a**) An ion aggregation system (from MD simulation) with both osmolytes (red balls) and waters (green balls). (**b**_**1**_) The graph or network representation is composed of only vertices (0-simplex) and edges (1-simplex). (**b**_**2**_) The simplicial complex representation has higher order simplexes, including triangles (faces) and tetrahedrons (solids). (**c**_**1**_) The distribution of Eigenvalues from the adjacency matrixes from the graph model. (**c**_**2**_) The persistent barcodes computed from a persistent homology representation.

Essentially, simplicial-complex-based persistent homology models are very different from traditional graph or network models. In general, Laplacian matrixes or adjacent matrixes are constructed from graph models and their eigen spectrum information is used in structure characterization. In contrast, persistent homology describes the structure with the topological invariants together with a geometric measurement. Figure 2 illustrates the comparison between persistent homology model and traditional graph models. The two types of models reveal very different topological information of the biomolecular systems.

We use notations *a*_*k*,*j*_ and *b*_*k*,*j*_ to represent birth times and death times of the *j*-th topological invariant of *k*-th dimension. The set of *k*-th dimensional barcodes is denoted as *L*_*k*_. The persistent Betti number (PBN)^66,88,93,96^ is defined as $f(x;{L}_{k})={\sum }_{j}\,{\chi }_{[{a}_{k,j},{b}_{k,j}]}(x)$. We propose two new functions, i.e., Boltzmann persistent entropy and Persistent radial distribution function.

#### Boltzmann persistent entropy

The persistent entropy has been proposed^96–99^ to measure the system disorder. For the *k*-th dimensional barcodes, it is defined as,

1 $${S}_{k}=\mathop{\sum }\limits_{j}^{{N}_{k}}\,-\,{p}_{k,j}\,\mathrm{ln}({p}_{k,j}),\,k=0,1,2.$$

with the probability function,

2 $${p}_{k,j}=\frac{{b}_{k,j}-{a}_{k,j}}{{\sum }_{j}^{{N}_{k}}({b}_{k,j}-{a}_{k,j})},\,k=0,1,2;j=1,2,3,\mathrm{...}.,{N}_{k}.$$

Even though persistent entropy has been a powerful tool for the characterization of “topological disorder”, its physical meaning is usually unclear, thus hinders its further application in chemical, physical and biological systems. In this paper, we propose a Boltzmann persistent entropy (BPE) based on the Betti energy and Boltzmann distribution. Essentially, we define a Betti energy for the *j*-th number of *k*-dimension Betti bar as follows,

3 $${E}_{k,j}=\alpha {\left(\frac{{b}_{k,j}-{a}_{k,j}}{\eta }\right)}^{\kappa },\,k=0,1,2;j=1,2,3,\mathrm{...}.,{N}_{k}.$$

Here *κ* is an integer, *η* is a scale value with the same unit of the filtration parameter, and *α* is an energy-related constant value. The probability function is then defined according to the Boltzmann distribution,

$${p}_{k,j}=\frac{{e}^{-\frac{{E}_{k,j}}{{k}_{B}T}}}{{\sum }_{j}^{{N}_{k}}{e}^{-\frac{{E}_{k,j}}{{k}_{B}T}}},\,k=0,1,2;j=1,2,3,\mathrm{...}.,{N}_{k}.$$

Here *k*_*B*_ is the Boltzmann constant and *T* is the thermodynamic temperature. The BPE can then be calculated from Eq. (1). Physically, when a Betti bar has a longer length, it will contribute a larger Betti energy, thus a lower probability. In contrast, a longer Betti bar has a higher probability in the traditional persistent entropy. Note that a long persisting *β*_0_ bar always exists in *β*_0_ barcodes. In traditional persistent entropy, the probability value for this long persisting bar is exactly equal to 1.0, and persistent entropy is always equal to 0 irrespective of the other *β*_0_ bars, if this long persisting bar is considered in persistent entropy. In our PBE, this bar contributes zero Betti energy according to Eq. (3), thus a probability zero. Note that in our calculation below we take *α* = *k*_*B*_*T*, *η* = 1 Å and *κ* = 2.

#### Persistent radial distribution function

Based on the *β*_0_ barcodes, we propose the persistent radial distribution function (PRDF) as follows,

4 $$f(x;{L}_{0})=\frac{{x}_{t}}{{N}_{0}}\sum _{j}\,\frac{\delta (x-{b}_{0,j})}{4\pi {x}^{2}}.$$

Here *x*_*t*_ is the filtration value when the PBN reduces to one, i.e., only one connected component. The integer *N*_0_ is the total number of *β*_0_ bars. Essentially, if we consider the global interactive persistent homology, our PRDF will result in the conventional radial distribution function^100^. On the other hand, If we use the local interactive persistent homology, our PRDF will focus on the interaction within each cell of the Voronoi diagram. A more detailed discussion is given in Section 1.2.2.

### Weighted persistent homology

The weighted persistent homology models have been proposed to incorporate physical, chemical and biological properties into topological modeling^53^. They can also be designed to characterize local topological information and certain special interaction patterns. In this paper, we will focus on two WPH models, i.e., localized persistent homology and interactive persistent homology.

#### Localized persistent homology

The design of our LPH model is inspired by the great success of element specific persistent homology (ESPH)^43,44^. Different from all previous topological models, which consider the data/structure as an inseparable system, ESPH decomposes the data/structure into a series of subsets made of certain type(s) of atoms, which have been found to characterize very well various biological properties, such as hydrophobic or hydrophilic interactions^43–49^. Moreover, our LPH model is very different from persistent local homology^92,101–105^. Mathematically, persistent local homology studies the relative homology groups between a topological space and its subspace, while LPH explores the homology groups from local topology. Previously, LPH has been used to characterize local topological features of biomolecular structure or complexes^53^. In LPH, the structure is decomposed into a series of local domains or regions, that may overlap with each other, and persistent homology analysis is then systematically applied on part (or all) of these local domains or regions. In this paper, our main focus is to characterize the local features, such as ion clustering, double layer and local aggregations, that widely exists in ion or molecular aggregation and hydrogen-bonding networks.

**Figure 3:**
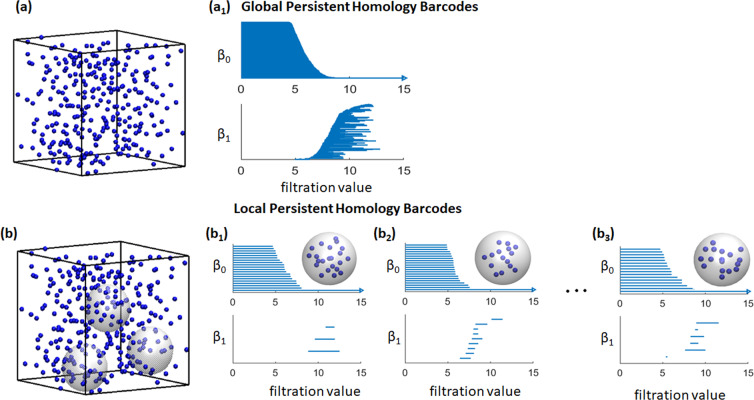
Illustration of the global and localized persistent homology analysis. The corresponding persistent barcodes for (**a**) Global and (**b**) Local approaches are demonstrated. The global persistent homology considers all the molecules in the simulation box as shown in (**a**), while localized persistent homology is carried out within each local region shown as grey spheres in (**b**). The persistent barcodes corresponding to three such local regions are illustrated from (**b**_**1**_)–(**b**_3_). In our LPH, we systematically consider all the molecules and generate a local region (of the same size) for each of them.

Mathematically, the global persistent homology analysis considers the complete domain, while the localized persistent homology is performed on a local region, subdomain or subspace. Note that topological invariants for the global structure is not simply the addition of all local invariants. Stated differently, topological invariants are usually not additive! In the current paper, we define the subspace as a sphere with radius (*R*_*c*_). More specifically, a sphere of radius *R*_*c*_ is considered around each molecule (either osmolyte or water molecule) and only the molecules within this sphere are chosen for the localized persistent homology analysis. Figure 3 illustrates the persistent homology analysis performed on molecular dynamics simulation data using two different approaches. Figure 3(a) and a(1) show the osmolyte distribution and their corresponding persistent barcodes obtained from persistent homology analysis. Figure 3(b) depicts the way of selecting local regions. Essentially, an individual molecule is selected and a sphere of radius *R*_*c*_ is drawn around it. All molecules within this enclosure are chosen as its local neighbors. The persistent homology analysis is carried out for all the selected molecules to generate the local persistent barcodes. This procedure is repeated for each molecule in the configuration. The corresponding persistent barcodes are as shown in Fig. 3(b1) to (b3). In essence, each molecule in a given configuration is associated with certain local neighbors which determine its local structure.

#### Interactive persistent homology

The interactive persistent homology (IPH) was proposed to study the interaction between proteins and ligands^46^. The essential idea is to study the topological invariants of the interaction networks, which are formed between protein atoms and ligand atoms. More specifically, for a protein-ligand complex, an interaction matrix can be built with its elements as the Euclidean distance between two atoms. However, if two atoms come from the same molecule (either protein or ligand), its distance is set to infinity, meaning they will never interact in IPH. In this way, the IPH model can be used in the characterization of the protein-ligand interactions. Actually, IPH based machine learning models are found to deliver the best results in protein-ligand binding affinity prediction^46,47,49^. Note that it seems to be better called as interaction persistent homology, as the model studies the interactions.

In this section, we use IPH models to characterize the interactions between osmolyte and water molecules. Two different models, i.e., global-scale IPH and local-scale IPH, are considered. In global-scale model, when an osmolyte molecule is selected, the distances (*d*_*ij*_) between all the water molecules in the domain to this osmolyte molecule are considered. More specifically, suppose there are *N*_*w*_ number of water molecules, a global-scale IPH matrix of size (*N*_*w*_ + 1) × (*N*_*w*_ + 1) can be constructed between a selected osmolyte molecule and all water molecules as follows,

5 $${M}_{ij}=\{\begin{array}{ll}{d}_{ij}, & {\rm{if}}\,{T}_{ype}(i)\ne {T}_{ype}(j);\\ \infty , & {\rm{otherwise}}\end{array}$$

Here *T*_*ype*_(*i*) is used to tell if the *i*-th molecule is osmolyte or water, i.e., type of the molecule. If there are *N*_*s*_ number of osmolyte molecule, we can construct a total *N*_*s*_ number of global-scale IPH matrices, with size (*N*_*w*_ + 1) × (*N*_*w*_ + 1). From these matrices, PRDF as in Eq. (4) can be calculated and the average of these PRDFs will characterize the same physical properties as the traditional radial distribution function^100^.

**Figure 4:**
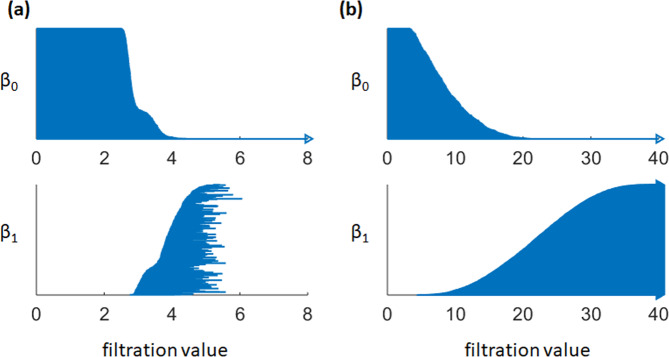
The persistent barcodes obtained using persistent homology and (**b**) interactive persistent homology. Note that in interactive persistent homology, interactions happen only between two types of different molecules. That is to say, water molecules can only interact with osmolyte molecules and vice versa. No interactions exist between water and water, or between osmolyte and osmolyte. It can be seen that the *β*_1_ bars generated from an interactive persistent homology remain persistent forever.

In local-scale IPH, a similar IPH matrix as in Eq. (5) is considered. But this new IPH matrix is now of size (*N*_*w*_ + *N*_*s*_) × (*N*_*w*_ + *N*_*s*_), meaning all distances between water and osmolyte molecules are considered simultaneously. The new IPH matrix based filtration characterizes dramatically different topological information. More specifically, molecules with shorter distances to their neighbors will form connections at earlier stage of the filtration. In this way, a Voronoi diagram will naturally form when water molecules connect to their center osmolyte molecule. Later, Voronoi cells will merge with closest neighbors to become a well-connected entity. The *β*_0_ barcodes capture very well the above topological information. And the corresponding PRDFs describe the local interactions within the Voronoi cells. A comparison of the persistent barcodes obtained from persistent homology and interactive persistent homology is illustrated in Fig. 4. It can be seen that they show totally different patterns.

Essentially, each osmolyte molecule can interact directly with all water molecules in global-scale model and the resulting PRDF (from *β*_0_ barcodes) characterizes the same physical properties as radial distribution function. In local-scale IPH model, only the interactions between the osmolyte molecule and water molecules in its Voronoi cell, and the Voronoi cell-cell interactions are captured in *β*_0_ bars. It should be noticed that the corresponding PRDFs only describe the local interaction information, and they are very different from the traditional radial distribution function. It should also be noticed that the value for our local PRDFs will decrease to zero when the filtration size is large enough.

### WPH for osmolyte molecular aggregation and hydrogen-bonding network analysis

The weighted persistent homology models are considered for the study of topological structures of two types of osmolytes, namely, trimethylamine N-oxide (TMAO) and urea. Two models, i.e., localized persistent homology and interactive persistent homology, are used to reveal the local topological features in the ion aggregation, hydrogen-bonding networks and their interactions. Note that only Vietoris-Rips complex is used in all our persistent homology models.

#### MD simulation and data generation

The molecular trajectory or the time evolution data of the two osmolytes needed for the current work is generated using a molecular dynamics simulation. We consider the same molecular dynamics (MD) setting as in the paper^31^. More specifically, we consider GROMACS-5.1.2^106,107^ for the MD simulation. The four point (TIP4P-EW)^108^ water model is used, and Kast model^109^ is adopted for TMAO whereas the urea model is from AMBER package^110^. Two osmolytes with eight different concentrations, from 1 M to 8 M, in pure water are studied, respectively. To construct the initial state, urea/TMAO molecules are randomly distributed using insert-molecules utility in GROMACS, after that 3000 water molecules are inserted randomly into the cubic simulation box. We carry out the equilibration process under NVT conditions (Temperature = 300 K) for 10 ps and then under NPT conditions for 100 ps using 2 fs time step, Berendsen thermostat *τ* = 0.1 ps) and barostat (*τ* = 2 ps). LINCS algorithm^111^ is used for bonds and the angles constriction. Further, we carry out three repeats under NPT conditions for 100 ns with Berendsen thermostat (Temperature = 300 K, *τ* = 0.1 ps), Parrinello-Rahman barostat (Pressure = 1 bar, *τ* = 2 ps) and using a time step of 2 fs. The integration of Newton’s equation of motion is done by using a leap-frog algorithm. A cut-off of 1.0 nm is used for both van der Waals (VDW) interaction and short-range electrostatic interaction. Particle mesh Ewald (PME)^112^ method is employed to deal with the long-range electrostatic interactions. The configuration trajectories are output every 1 ps.

#### LPH analysis of osmolytes

The localized persistent homology is used to explore the topological fingerprints of molecular aggregation and hydrogen-bonding network at a local scale. In TMAO systems, there are 63, 125, 204, 290, 400, 533, 700 and 887 TMAO molecules with 3000 water molecules from concentration 1 M to 8 M, respectively. In urea systems, there are 60, 120, 192, 267, 352, 450, 555 and 681 urea molecules with 3000 water molecules for concentration 1 M to 8 M respectively. To analyze the local topology in their molecular aggregation and hydrogen-bonding networks, the TMAO and urea molecules are coarse-grained as their nitrogen and carbon atoms, respectively. The water molecules are coarse-grained as their oxygen atoms. Since the configuration data is obtained from an NPT simulation, the size of simulation box is allowed to adjust for each configuration to attain equilibrium conditions. Periodic boundary condition is used in the specification of local domains. For each simulation, we consider 101 frames (or configurations) sampled at equal intervals from the simulation trajectory. Our topological analysis is performed on these 101 frames.

In our LPH model, a local spherical region is defined for each ion using a cutoff radius *R*_*c*_ and the atoms within this enclosure is chosen for analysis. Persistent homology is applied to each of these local regions and the persistent barcodes are computed. We used an open source software Ripser^113^ for the computation of persistent barcodes. The persistent Betti functions (PBNs) and the Boltzmann persistent entropy (BPEs) are calculated from these barcodes.

### IPH analysis of osmolytes

Both global-scale and local-scale IPHs are considered for analyzing the interactions between osmolyte molecules and water molecules. In global-scale IPH, for each osmolyte molecule, we can construct a series of IPH matrixes as in Eq. (5) with the same size of 3001 × 3001, as there are totally 3000 water molecules. From the *β*_0_ barcode of the IPH matrices, a single PRDF can be calculated. Further by averaging the PRDFs over all the 101 frames and all osmolyte molecules in each frame, we can obtain the average global-scale PRDF.

In local-scale IPH, for each configuration or frame, an individual IPH can be constructed. Note that the size of the local-scale IPH matrix as in Eq. (5) is (*N*_*w*_ + *N*_*s*_) × (*N*_*w*_ + *N*_*s*_), i.e., the total number of osmolyte and water molecules in the simulation. The average local-scale PRDF (or PBN) can be evaluated by averaging their values over all the 101 frames.

## Results and discussions

In this section, we systematically study the local topological features and interaction properties of the osmolyte molecular aggregation and their hydrogen-bonding networks. The corresponding PBNs, BPEs and PRDFs are used to quantitatively characterize the intrinsic local topology information.

### LPH for molecular aggregation and hydrogen-bonding networks

**Figure 5:**
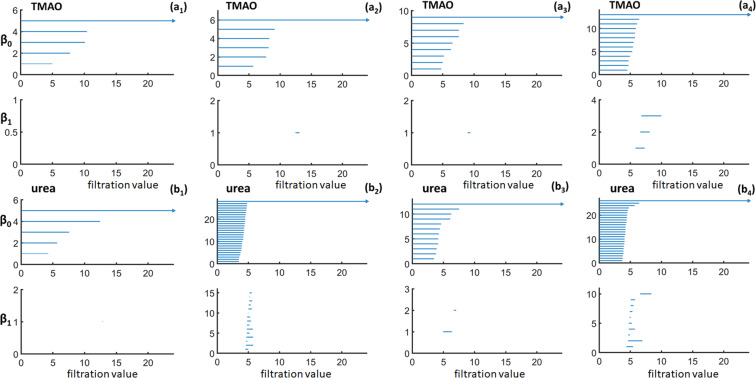
The local persistent barcodes for TMAO and urea aggregation. TMAO and urea molecules are coarse-grained as their nitrogen and carbon atoms. Subfigures (***a***_**1**_ to ***a***_**4**_) represent the results from TMAO with concentration 2 M, 4 M, 6 M, and 8 M, respectively. Subfigures (***b***_**1**_ to ***b***_**4**_) represent the results from urea with concentration 2 M, 4 M, 6 M, and 8 M, respectively. The barcodes are generated from a randomly picked molecule at the last frame of the simulation. A cutoff radius of 9 Å is used. Roughly speaking, both *β*_0_ and *β*_1_ bars tend to increase with the concentration.

To facilitate an intuitive understanding of local topological information of molecular aggregation, we demonstrate the persistent barcodes calculated from TMAO and urea systems with a cutoff radius of *R*_*c*_ = 9 Å. More specifically, we consider the last configuration of the MD simulation from four different concentrations. An osmolyte molecule is randomly chosen from the last frame and its neighbouring osmolyte molecules located within the cutoff radius *R*_*c*_ = 9 Å are selected. Persistent homology analysis is then applied on these molecules. The results from TMAO and urea systems are demonstrated in Fig. 5. The indexes (a) and (b) corresponds to TMAO and urea respectively. The subscripts 1–4 indicates the four different concentrations considered, i.e., 2 M, 4 M, 6 M, and 8 M respectively. In both TMAO and urea, the total number of *β*_0_ bars roughly increase with concentration (M), indicating the aggregation of neighboring molecules with the concentration. The *β*_1_ bars also seem to appear more and more frequently with the increase in concentration.

The results shown in Fig. 5 are based on a randomly chosen molecule in the last frame of the simulation trajectories and can not characterize the overall behavior very well. To have a better comparison, we consider the ensemble average. Meaning, for each frame, the local persistent barcode from each molecule is calculated and then averaged. It should be noticed that we use the periodic boundary condition to include all the “neighboring” molecules. This process is repeated for all the 101 frames in each trajectory. We represent each persistent barcode as their PBN and BPEs. These PBNs are then averaged over all the frames and all the molecules in each frame to generate a single PBN for each simulation or trajectory. The BPEs are averaged over all the molecules in each frame, so that a total 101 BPEs are obtained from each simulation.

**Figure 6:**
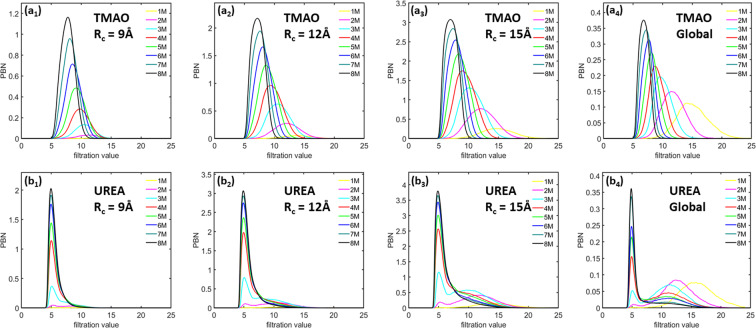
The comparison of average *β*_1_ PBNs for (**a**) TMAO and (**b**) urea at eight different concentrations from 1 M to 8 M. Subfigures (***a***_**1**_ to ***a***_**3**_) are results from TMAO with three different cutoff radii namely., *R*_*c*_ = 9 Å, 12 Å and 15 Å, respectively. Subfigures (***b***_**1**_ to ***b***_**3**_) are results from urea with three different cutoff radii namely., *R*_*c*_ = 9 Å, 12 Å and 15 Å, respectively. The *β*_1_ PBNs are averaged over all the frames and all molecules in each frame. Subfigures (***a***_**4**_) and (***b***_**4**_) are the PBNs obtained from a global persistent homology analysis. It can be seen that, TMAO and urea show dramatically different local topological characteristics.

Figure 6 shows the *β*_1_ PBNs obtained for the TMAO and urea system at eight different concentrations, from 1 M to 8 M, using three different cutoff radii. The indexes (a) and (b) corresponds to TMAO and urea respectively. The subscripts 1–3 indicates the three different cutoff radii namely., *R*_*c*_ = 9 Å, 12 Å and 15 Å respectively. The corresponding global persistent homology analysis for TMAO and urea is shown in (a_4_) and (b_4_) respectively. As stated above, *β*_1_ bars represent the ring, circle and loop structures in the system. For TMAO system, at each cutoff radius, the peak value of the local *β*_1_ PBNs systematically increases with the concentration, indicating that more and more circle structures are generated. At the same time, the position of these peak values shifts from around 13 Å to 7 Å, which implies a systematic decrease in the size of these circles. When we consider larger cutoff radii, similar topological patterns are observed. However, the peak values of PBNs from lower concentration systems increase much faster, even though all PBN peak values increase with the cutoff radius. This result indicates that for a lower concentration system, there exists large-sized topological features which can not be well characterized by LPH with a small cutoff radius. For urea system, their PBNs have a dramatically different behavior in comparison with TMAO. Roughly speaking, there are two types of peak for urea system, especially the urea system at lower concentrations. One type of peak is located around 5 Å, and the other is around 10 Å to 12 Å. The peak at 5 Å appears even at very low concentrations and its magnitude keeps increasing with the concentration rise. The shape of this peak is much narrower than that of TMAO PBNs. The second type of peak can only be distinctly observed at lower concentrations. It has much smaller magnitude compared with that of the first type of peak.

From Fig. 6, we can also see that TMAO and urea demonstrate dramatically different local topological characteristics. Essentially, TMAO shows a regular local network structure. The size and total number of the circle structures from these networks consistently decrease and increase with the concentration, respectively. In contrast, urea shows a cluster-like local aggregations. Urea molecules form local clusters, whose size stays relatively consistent but the total number consistently increases with concentration. More interestingly, if we compare our LPH results with the ones from persistent homology analysis of the whole osmolyte systems^31^, as in Fig. 6(a4) and (b_4_), we can find some unique similarities and differences. Essentially, the general pattern of PBNs at lower filtration values has less changes and remains relatively stable, while PBNs at larger filtration values change more dramatically. Stated differently, the LPH focuses more on the local topological information and systematically attenuates the influence from global topological features.

**Figure 7:**
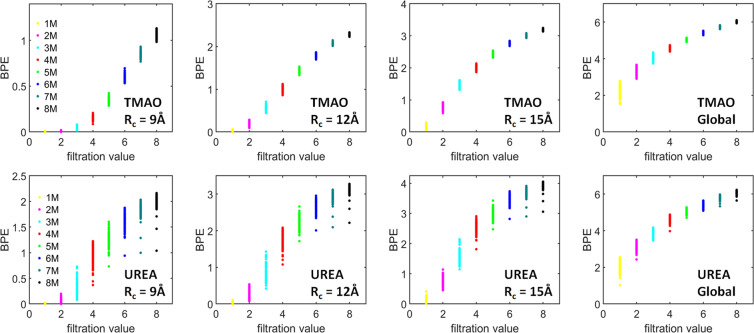
The comparison of average persistent entropies for (**a**) TMAO and (**b**) urea at eight different concentrations from 1 M to 8 M. Subfigures (***a***_**1**_ to ***a***_**3**_) are results from TMAO with three different cutoff radii namely., *R*_*c*_ = 9 Å, 12 Å and 15 Å, respectively. Subfigures (***b***_**1**_ to ***b***_**3**_) are results from urea with three different cutoff radii namely., *R*_*c*_ = 9 Å, 12 Å and 15 Å, respectively. Subfigures (***a***_**4**_) and (***b***_**4**_) are the PEs for TMAO and urea obtained from global persistent homology analysis, respectively. The BPEs are averaged over all the molecules in each frames, thus a total 101 BPEs are obtained for each simulation. It can be seen that, for a small cutoff radius of *R*_*c*_ = 9 Å, both TMAO and urea BPEs at 1 M are almost all zero. Further, the average BPEs for both systems increase with the concentration, but their BPE variances have very different properties. The TMAO BPE variance decreases with concentration while urea BPE variance increases.

Other than the PBNs, we can also calculate BPEs from the LPH barcodes and use them to characterize the “topological regularity”. Figure 7 demonstrates the *β*_1_ BPEs for both TMAO and urea at eight concentrations and three cutoff radii as stated above. Note that for each simulation or trajectory, we consider 101 configurations or frames which generates 101 *β*_1_ BPEs. It can be seen that, at a small cutoff radius, the BPE values from 1 M concentration is almost all zeros, meaning that there is almost no circle structures at local scale. This is consistent with the PBN profile in Fig. 6. Further, the average BPE value increases systematically with the concentration for both TMAO and urea. However, the BPE variance shows a very different behavior. With the concentration increase, the TMAO BPE variance systematically decreases, while urea BPE variance consistently increases. These results are also consistent with our findings from persistent homology analysis of the whole system^31^ and is also presented here for clarity. Essentially, with the concentration increase, all osmolyte systems become topologically more and more disordered. However, the variation of topological regularity for each trajectory decreases in the TMAO system but increases in the urea system. The BPE are found to be consistent with the global persistent homology analysis as shown in Fig.7(a4) and (*b*_4_) for TMAO and Urea respectively.

**Figure 8:**
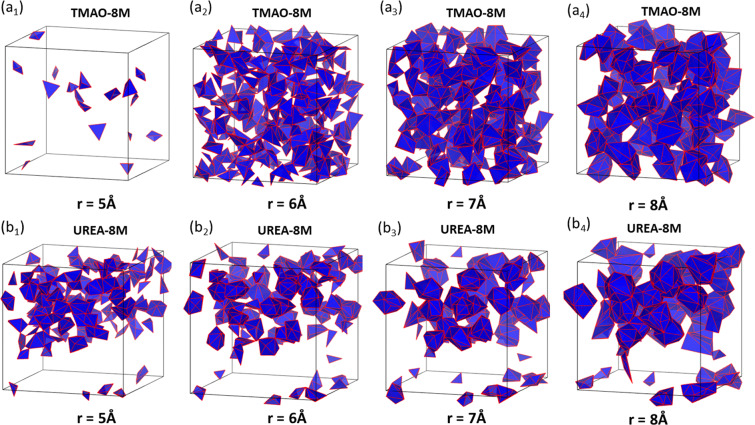
The comparison of the simplicial complexes from TMAO and urea molecule aggregation at 8 M concentration. The subfigures (***a***_**1**_ to ***a***_**4**_) are for TMAO and subfigures (***b***_**1**_ to ***b***_**4**_) are for TMAO. The filtration for (**a**_**1**_ to **a**_**4**_) is 5 Å, 6 Å, 7 Å and 8 Å, respectively. Note that we only plot the 2-simplexes and 3-simplexes for better visualization. The same setting is used for urea systems in ***b***_**1**_ to ***b***_**4**_.

To have an intuitive understanding of the inner topological differences between TMAO and urea molecular aggregation, we generate simplicial complexes from the last frame of the simulation data of TMAO and urea at highest concentration (8 M). For a better visualization, we consider the value of filtration parameter *r* to be 5 Å, 6 Å, 7 Å and 8 Å, and plot only the 2-simplexes (triangles) and 3-simplexex (tetrahedrons). The results are illustrated in Fig. 8. It can be seen that, TMAO molecules are more evenly distributed, while urea molecules tend to concentrate into clusters. Topologically, evenly-distributed molecules will generate more “large” circle structures (longer bars in *β*_1_ barcodes), while local clustering contributes more small circles (shorter bars in *β*_1_ barcodes).

#### LPH based topological features of hydrogen-bonding networks

In our hydrogen-bonding network analysis, we consider the topological features for water molecules at a local scale. Similar to osmolyte systems, The LPH analysis is carried out for each water molecule along with its neighbours located within a cutoff radius *R*_*c*_. For each frame, we systematically go over all the 3000 water molecules and calculate 3000 local persistent barcodes. Again periodic boundary condition is considered to include all “neighboring” water molecules. The process is repeated over all the 101 frames in each trajectory. A single *β*_1_ PBN is generated for each simulation by averaging *β*_1_ PBNs over all the 101 frames and all the 3000 water molecules in each frame. The *β*_1_ BPEs are averaged over the 3000 water molecules in each frame, so that a total 101 *β*_1_ BPEs are obtained from each simulation. Two cutoff radii, i.e, *R*_*c*_ = 7 Å and *R*_*c*_ = 9 Å, are considered in our LPH analysis.

**Figure 9:**
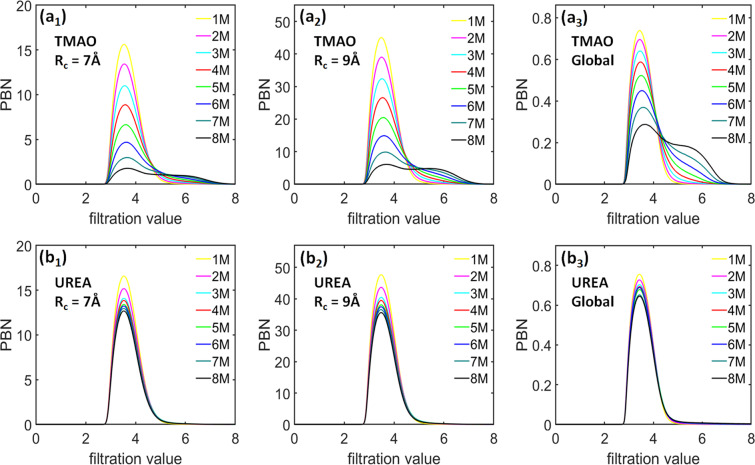
The comparison of average *β*_1_ PBNs for hydrogen bonding networks of (**a**) TMAO and (**b**) urea using two different cutoff radii at eight different concentrations from 1 M to 8 M. Subfigures (***a***_**1**_ to ***a***_**2**_) are results from TMAO hydrogen bonding networks with two different cutoff radii namely., *R*_*c*_ = 7 Å and 9 Å, respectively. Subfigures (***b***_**1**_ to ***b***_**2**_) are results from urea hydrogen bonding networks with two different cutoff radii namely., *R*_*c*_ = 7 Å and 9 Å, respectively. Subfigures (***a***_**3**_) and (***b***_**3**_) are the corresponding PEs obtained from global persistent homology analysis. The coarse-grained representation of water as its oxygen atom is considered. The indexes (**a**) and (**b**) corresponds to TMAO and urea respectively. The PBNs are averaged over all the molecules and configuration numbers. It can be seen that, TMAO and urea show very different topological characteristics.

Figure 9 shows the comparison of average *β*_1_ PBNs for TMAO and urea hydrogen-bonding networks. Figure 9(*a*_1_) and (*a*_2_) indicates that, for TMAO system, the PBNs have a peak value located at around 3.5 Å. With the concentration increase, the peak value of TMAO PBNs gradually decreases. In the meantime, there is a consistent rise of the PBN values in the range from around 4.5 Å to 7.0 Å. Even though all PBNs significantly increase with the cutoff radius, the general PBN profile pattern from eight different concentrations is highly consistent. Similar to TMAO, Fig.9(*b*_1_) and (*b*_2_) shows that, urea PBNs also have a peak value at filtration value 3.5 Å. The peak value slightly decreases with the concentration increase. Further, the general PBN profile pattern from eight different concentrations shares a remarkable similarity at two different local scales, even though the PBN peak values are systematically increased.

From Fig. 9, we can see that TMAO and urea hydrogen-bonding networks demonstrate dramatically different local topological characteristics. For TMAO hydrogen-bonding networks, with the concentration increase, there is a systematic decrease of small-sized circle structures as well as an increase of relatively large-sized circle structures. For urea hydrogen-bonding networks, there is only a slight decrease of small-sized circle structures and no significant increase of large-sized circle structures. More interestingly, if we compare our LPH results with the ones from the whole hydrogen-bonding network in both ion and osmolyte systems^31,50^, we can see that there exists a great similarity in their PBNs. Essentially, TMAO and urea hydrogen-bonding networks show two types of topological behaviors. With the concentration increase, TMAO molecules tend to destroy the local hydrogen-bonding networks, resulting in a significant increase of the large circle structures. In contrast, the urea molecules have a much less impact on the hydrogen-bonding networks.

**Figure 10:**
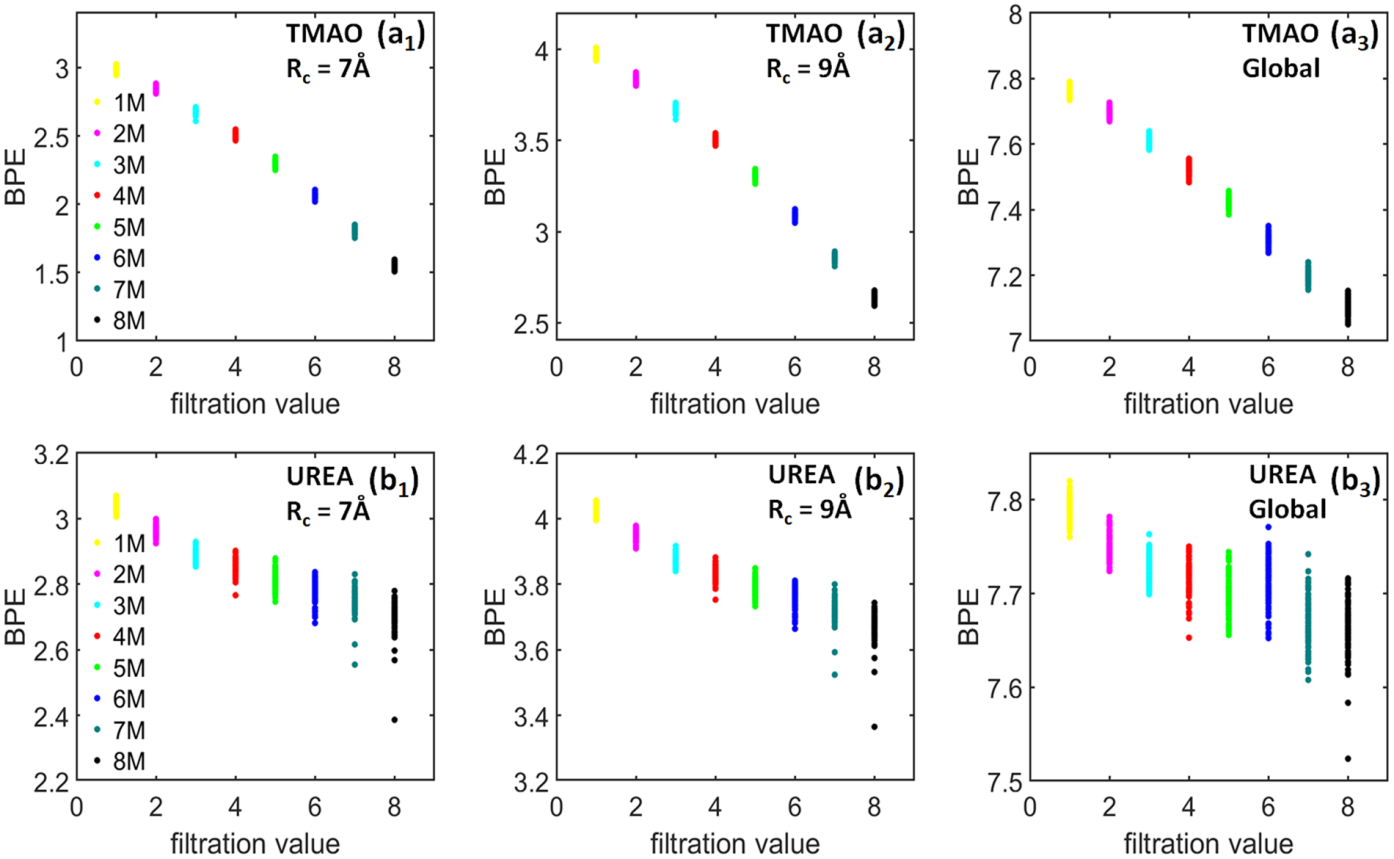
The comparison of average *β*_1_ BPEs for hydrogen-bonding networks from (**a**) TMAO and (**b**) urea systems using two different cutoff radii at eight different concentrations from 1 M to 8 M. Subfigures (***a***_**1**_ to ***a***_**2**_) are results from TMAO hydrogen bonding networks with two different cutoff radii namely., *R*_*c*_ = 7 Å and 9 Å, respectively. Subfigures (***b***_**1**_ to ***b***_**2**_) are results from urea hydrogen bonding networks with two different cutoff radii namely., *R*_*c*_ = 7 Å and 9 Å, respectively. The BPEs are averaged over all the water molecules in each frames, thus a total 101 BPEs are obtained for each simulation. It can be seen that, the average BPE decreases with the concentration for both TMAO and urea. However, the BPE variance for urea systematically increases. (***a***_**3**_) and (***b***_**3**_) are the PEs obtained from a global persistent homology analysis.

The persistent entropy from the LPH barcodes can also be used to characterize the “topological regularity” of hydrogen-bonding networks. Figure 10 demonstrates the *β*_1_ BPEs for both TMAO and urea hydrogen-bonding networks at eight concentrations and two cutoff radii. The indexes (a) and (b) denote TMAO and urea systems respectively, at eight different concentrations from 1 M to 8 M. The subscripts 1–2 indicates the cutoff radii *R*_*c*_ = 7 Å and *R*_*c*_ = 9 Å respectively. Similar to molecular aggregation analysis, for each simulation, we consider 101 configurations or frames and generate 101 *β*_1_ BPEs. It can be seen that, the average BPE value for both TMAO and urea hydrogen-bonding networks decreases with the concentration increase. The same pattern is observed at two local scales. Topologically, these results indicate that both the hydrogen-bonding networks become more and more regular and lattice-like with concentration increase. Note that molecular aggregation has a totally different topological behavior, their BPE value systematically increases with the concentration. More interestingly, the urea BPE variance is significantly larger than that of TMAO and consistently increases with the concentration. This is exactly the same as in the urea aggregation system.

In summary, we have used LPH models to explore the osmolyte molecular aggregation and their hydrogen-bonding networks. Essentially, we segregate osmolyte molecules from water molecules, and study their local topological features separately. In the next section, we will focus on the interaction between osmolyte molecules and water molecules and characterize the topology of their interaction networks.

**Figure 11:**
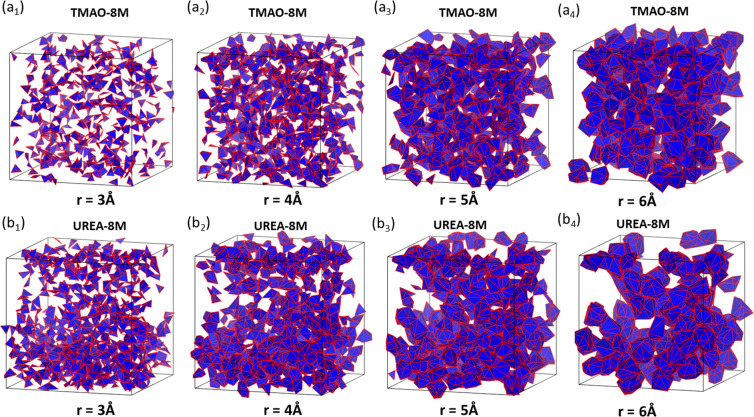
The comparison of the simplicial complexes from hydrogen-bonding networks from TMAO and urea molecules at 8 M concentration. The subfigures (***a***_**1**_ to ***a***_**4**_) are for TMAO and subfigures (***b***_**1**_ to ***b***_**4**_) are for TMAO. The filtration values for (**a**_**1**_ to **a**_**4**_) is 3 Å, 4 Å, 5 Å and 6 Å, respectively. Note that we only plot the 2-simplexes and 3-simplexes for better visualization. The same setting is used for urea systems in ***b***_**1**_ to ***b***_**4**_.

Figure 11 illustrates the simplicial complexes of the hydrogen-bonding networks from TMAO and urea. They are generated from the last frame of the simulation data of TMAO and urea at highest concentration (8 M). We consider the value of filtration parameter *r* to be 3 Å, 4 Å, 5 Å and 6 Å, and plot only the 2-simplexes (triangles) and 3-simplexex (tetrahedrons), for a better visualization. It can be seen that, similar to the results in Fig. 8, water molecules in TMAO systems are more evenly distributed, while water molecules in urea sytems tend to concentrate into clusters. Topologically, evenly-distributed water molecules will generate more “large” circle structures (longer bars in *β*_1_ barcodes), while local clustering contributes more small circles (shorter bars in *β*_1_ barcodes). For the above analysis, it can be noticed that persistent barcode provides a unique way of analyzing the inner topological structures of the systems.

### IPH based topological features for osmolyte-water interaction network

**Figure 12:**
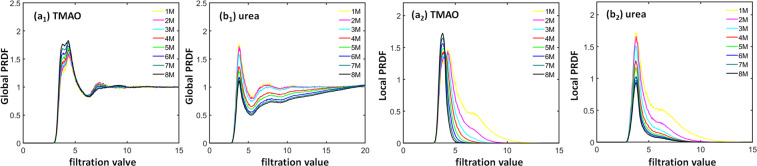
The comparison of global-scale and local-scale PRDFs for (**a**) TMAO and (**b**) urea. Subfigures (***a***_**1**_ to ***a***_**2**_) are results from global-scale and local-scale PRDFs of TMAO systems, respectively. Subfigures (***b***_**1**_ to ***b***_**2**_) are results from global-scale and local-scale PRDFs of urea systems, respectively. The PRDF of N-O is examined in the case of TMAO and C-O for the urea osmolyte. It can be seen that, the first peak value of the TMAO PRDFs increases with the concentration, while the first peak value of urea PRDFs decreases with the concentration.

Figure 12 shows the comparison of global-scale and local-scale PRDFs for both TMAO and urea systems. The indexes (a) and (b) represents TMAO and urea respectively. The subscripts 1–2 corresponds to the global-scale and local-scale PRDFs respectively. Both global-scale and local-scale PRDFs are normalized with the number density of the oxygen atom. The number density in global-scale is calculated by considering the number of oxygen atoms averaged over all the spheres around each ion with radius *r*_*max*_. The value of *r*_*max*_ is half the box size. In the local-scale, the number density is simply the number of oxygen atoms divided by the volume of the simulation box for a given concentration. Essentially, the global-scale PRDFs are identical to the traditional radial density functions. It can be seen that both TMAO and urea have two very obvious peaks, one located at around 4 Å and the other located at around 7 Å. However, their behaviors are dramatically different. For TMAO, the first peak value consistently increases with the concentration while the second peak value decreases with the concentration. The change of the TMAO peak values are relatively small, especially for the second peak. In contrast, both peaks of urea PRDFs vary greatly with concentration change. In local-scale IPH, PRDF values converge quickly to zero when the filtration value is larger than 12 Å, which is dramatically different from the situation in global-scale PRDFs when their values converge to 1 at large filtration value. However, the first peak of local-scale PRDFs has similar pattern as the global-scale ones. The TMAO peak value increases with concentration, while urea peak value decreases with concentration. Moreover, at the region of filtration value from 5 Å and 10 Å, which is the region for the second peak of global PRDFs, the TMAO PRDF values decrease much faster than those of urea. When the concentration is larger than 5 M, nearly all TMAO PRDF values drops to zero, while urea PRDF still remains largely positive.

**Figure 13:**
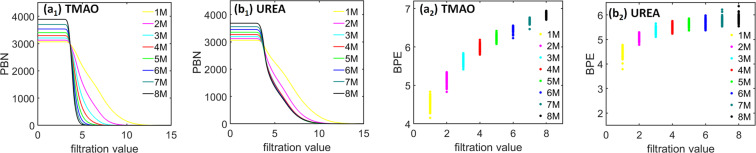
The comparison of average *β*_0_ PBNs and BPEs for hydrogen-bonding networks of (**a**) TMAO and (**b**) urea systems. Subfigures (***a***_**1**_ to ***a***_**2**_) are TMAO *β*_0_ PBNs and *β*_0_ BPEs, respectively. Subfigures (***b***_**1**_ to ***b***_**2**_) are urea *β*_0_ PBNs and *β*_0_ BPEs, respectively. The N-O pair interaction network is considered for analysis. The comparison of average *β*_0_ BPEs from the IPH analysis of TMAO and urea systems. For each configuration, a BPE value can be calculated, thus a total 101 BPEs are obtained for each simulation. It can be seen that, the average BPE increases with the concentration for both TMAO and urea. However, the BPE variance for urea systematically increases.

To have a better understanding of the local-scale PRDFs, we check the PBNs and PEs from the local-scale IPH. Figure 13 demonstrates *β*_0_ PBNs for TMAO and urea. The indices (a) and (b) represents TMAO and urea respectively. The subscripts 1–2 corresponds to the PBNs and PEs respectively. The *β*_0_ PBNs are directly related to PRDFs. It can be seen that indeed the TMAO *β*_0_ PBNs decrease much faster than those of TMAO at the filtration region from 5 Å to 10 Å, consistent with our observations in local-scale PRDFs. Further, we study the corresponding BPEs. It can be seen in Fig. 13, that the average BPE values for both local-scale IPH models increase with the concentration. More interestingly, the BPE variance for TMAO decreases with the concentration, while that for urea systematically increases with the concentration.

## Conclusion

In this paper, we use the weighted persistent homology to study the topological properties for osmolyte molecular aggregation and their hydrogen-bonding networks at a local scale. Two different models, i.e., localized persistent homology (LPH) and interactive persistent homology (IPH), are considered. We propose Boltzmann persistent entropy (BPE) to quantitatively characterize the topological features from LPH and IPH, together with persistent Betti number (PBN). Based on persistent barcodes, we have proposed the persistent radial distribution function (PRDF). It has been found that the global-scale PRDF will reduce to traditional radial distribution function. While local-scale PRDFs can efficiently characterize the local interactions within the Voronoi cells. We will consider MD simulations, including high pressure conditions, solution with both urea and TMAO, and protein with TMAO or urea, in our future works to fully elucidate the mechanisms for protein stabilization. Note that since any graph can be constructed into a simplicial complex (through clique complex), our weighted persistent homology models can be used in the analysis of graph and network structures from material, chemical and biological systems^114–116^.

## Data Availability

Our codes are available at https://www.ntu.edu.sg/home/xiakelin/WPH_Osmolytes.zip.
